# Loneliness, social isolation and ageing: a methodological approach to compare Latvian and Icelandic older populations in the course of COVID-19 pandemic

**DOI:** 10.21203/rs.3.rs-2870118/v1

**Published:** 2023-05-03

**Authors:** Ieva Reine, Madara Miķelsone, Helgi Guðmundsson, Andrejs Ivanovs, Signe Tomsone, Halldór S. Guðmundsson

**Affiliations:** Riga Stradiņš University; Riga Stradiņš University; University of Iceland; Riga Stradiņš University; Riga Stradiņš University; University of Iceland

**Keywords:** Survey of Health, Ageing and Retirement in Europe (SHARE), mental and physical health, marital status, financial, education, employment, gender

## Abstract

**Background:**

Feelings of loneliness and social isolation are common among the elderly, affecting both health and wellbeing. The COVID-19 pandemic has altered social connections through health precautions, restrictions and other factors. However, limited research has been conducted on how older people’s health and wellbeing in different countries has been impacted by the COVID-19 pandemic. The aim of this study was to develop methodology that would allow us to compare elderly populations, aged 67 + in Latvia and Iceland, and to discuss the potential impact of diverging factors on the association between loneliness, social isolation and health.

**Methods:**

Quantitative data on 420 respondents from Wave 8 of the Survey of Health, Ageing and Retirement in Europe (SHARE) was utilized in Latvia. Data on health and wellbeing of elderly in Iceland from a HL20 study with 1033 respondents was used to provide comparative analytic material for studying the differences between Latvia and Iceland, and within each country.

**Results:**

The study revealed considerable differences between the countries regarding the frequency of loneliness and social isolation. About 80% of Latvian respondents felt socially isolated and 45% were lonely, compared to 42.7% socially isolated and 30% lonely Icelanders. In general, more elderly people in Latvia experienced difficulties than their peers in Iceland. Social isolation tends to differ across genders and age groups in both countries. This is related to marital and employment status, financial situation, and education. COVID-19 had a stronger deteriorating effect on mental and physical health among both lonely Latvian and Icelandic respondents. However, health deterioration was stronger amongst more socially isolated Icelanders compared to Latvians.

**Conclusions:**

The study suggests that social isolation is a contributing factor and increases the risk of loneliness, which might have been enhanced by restrictions during the COVID-19 pandemic.

## Background

Loneliness and social isolation are common among older people and can be both negatively correlated with mental and physical health, although still relatively little is known about causal links [1]. The diversity of definitions and measurements, as well as different settings, complicates the use of the evidence for both researchers and policy makers. One way forward would be to pool the evidence from the literature on the drivers of loneliness and social isolation in old age and to study their impact on health, so that important domains and dimensions are measured [2] There is also a challenge in relation to interventions, as data are still very limited. To date, the evidence is not sufficient to determine whether modifying feelings of loneliness or social isolation levels will have an impact on subsequent health, also as regards the COVID-19 pandemic [3–5].

Healthy ageing, loneliness and social isolation are interconnected concepts [6–7]. Older adults are vulnerable to loneliness and social isolation, which can negatively impact their well-being [8]. This is a common issue facing elderly populations in many countries and can result from a variety of factors, including the loss of loved ones, decreased mobility and physical health, and limited access to social and community resources [9–10]. Both isolation and loneliness are found to be risk factors for a range of health outcomes [11–13]. Social isolation can have a negative impact on physical and mental health, leading to increased risk of chronic diseases, cognitive decline and mortality [14–16].

On the other hand, staying socially connected and engaged can help promote healthy ageing by improving overall well-being, reducing stress, and creating a sense of purpose [17–18]. The level of deprivation of social connectedness may depend on the quantity and/or quality of interactions with other people [19].

Studies have shown that older adults who participate in regular social activities and have strong relationships with family and friends are more likely to maintain better physical and cognitive health and experience less depression and loneliness [21].

This article is an outcome of the strategic EEA initiative, aiming to strengthen bilateral relations between Latvia and Iceland in a project called *Modelling of the Impact of Covid-19 on Public Health of Elderly People in Latvia and Iceland*. The project is expected to promote scientific and socioeconomically significant cooperation by using innovative methods that can help us understand and overcome the consequences of the COVID-19 crisis among the elderly. The current article is a starting point within the project that provides descriptive and comparative data for further evaluation of the scale of the impact of COVID-19, using statistical calculations of probability for several risk groups of the populations of Latvia and Iceland.

Latvia and Iceland are two countries that might have different levels of social isolation due to various factors such as culture, geography, population, and COVID-19 pandemic measures. In Latvia, the COVID-19 pandemic has impacted social connections, as measures such as lockdowns, and restrictions on gatherings have limited opportunities for in-person interactions [21]. The COVID-19 pandemic has had a similar impact on social interactions in Iceland as in Latvia, but the strong community ties may have helped mitigate the effects of social isolation. However, there is limited research comparing the levels of social isolation among older people in Latvia and Iceland.

In recent decades, longevity in Latvia has improved, with both men and women living longer than previous generations. Life expectancy at birth for Latvians in 2022 was 75.6 years [22]. Women have a longer life expectancy than men in Latvia, with women living an average of 80 years, while men live an average of 71 years. This shift has led to concerns about the social and economic consequences of an increasingly isolated and lonely population. Studies have suggested that loneliness in Latvia is linked to several factors, including changes in family structures, urbanization, and economic inequality. According to a survey by the Latvian Central Statistical Bureau in 2019 [23], around 45% of individuals aged 18 and above reported feeling lonely at least once per week, with those aged over 60 being most affected. Social support systems are another factor that can impact loneliness in Latvia. Social support from family and close relationships is often considered an integral part of Latvian culture. However, changes in family structures, such as higher divorce rates and migration to urban areas, have led to weakened familial relationships and diminished social support [24].

Also, Iceland’s demographics have undergone significant changes in recent years, including a growing and aging population, and an increase in immigration. Iceland has one of the highest life expectancy rates in the world, at an average of 81 years for men and 84 years for women [25]. This leads to an aging population, with individuals over 60 years making up around 21% of the population. The high rates of single-person households in Iceland have highlighted concerns about loneliness and social isolation among its citizens, particularly women and older adults. However, efforts to promote community involvement, public events, and digital support have been initiated to mitigate these issues [26–27].

To address healthy ageing issue, both Latvia and Iceland have implemented various programmes and initiatives aimed at promoting social engagement and combatting loneliness among the elderly. Several programs address the social isolation of older adults by providing social and healthcare services and more frequent home and web-based visits. Additionally, community-based programs aim to encourage people to participate in social activities and events to increase their level of social interaction and support. These can include social clubs and community centres, home visits and volunteer programs, and transportation services to help connect older people with community activities and events.

However, literature on interventions related to associations between social isolation, loneliness and health have generally received relatively little attention [8]. A recent Danish review of the effects of social isolation and loneliness [28]. confirms significant and beneficial effects for all age groups, regardless of whether the intervention was based on “social network enhancement”, “social support”, “social and emotional support” and “information and interview therapy and psychological work”.

Some factors that could impact levels of social isolation include cultural attitudes towards ageing, availability of community resources and support networks, and economic factors such as poverty and unemployment [29–30]. It is difficult to make a comparison between Latvia and Iceland without further information and analysis. Thus, research presented in this article will contribute to gaining more knowledge about the variations in wellbeing in both countries, and to discuss potential impact of diverging factors on the association between loneliness, social isolation and health.

## Method

### Data and population

We utilized quantitative data from Wave 8 of the Survey of Health, Ageing and Retirement in Europe (SHARE) [31–32], conducted in November 2019-March 2020 and June-August 2020 by doing 1089 computer-assisted telephone interviews. The questions examined how people over 50 years of age coped with socioeconomic and health-related impact of COVID-19. The sample for the current study consists of SHARE longitudinal panel participants in Latvia of age 67 and above– SHARE Wave 8 respondents, as well as those respondents in the Wave 8 update who were interviewed before the start of the COVID-19 pandemic. The sample size used in this study consists of 420 valid cases.

Quantitative data on health and wellbeing of the elderly in Iceland comes from a HL20 study [27] done in 2020 among individuals that had reached retirement age. An 1800-person simple random sample from the national registry was drawn among Icelandic citizens that had reached the age of 67. In total, 1033 persons answered the survey, either via a computer-assisted telephone interview (42%), or computer-assisted web interview (58%). The survey was conducted from November 2020 to January 2021.

### Variables

**Figure 1:**
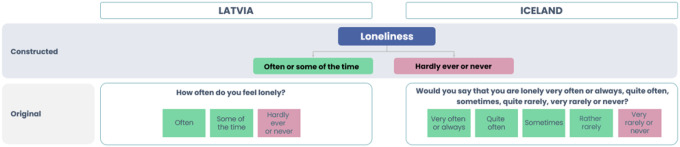
Categorisation of loneliness variables in Latvia and Iceland

**Figure 2:**
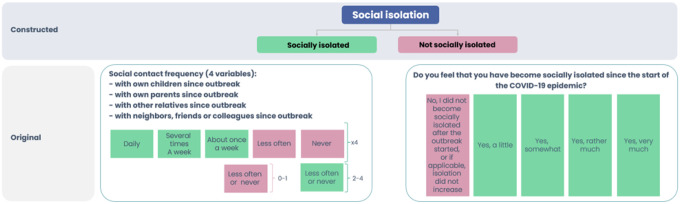
Categorisation of social isolation variables in Latvia and Iceland

To compare older populations in Latvia and Iceland, persons of age 67 and above were included in the analysis utilizing SHARE from Latvia like in the HL20 data from Iceland. Descriptive demographic variables – gender, age groups, marital status, education, employment, households’ ability to make ends meet/financial worries as well as health and wellbeing were standardized for comparative reasons. The response options for each variable were reviewed by the researchers and graphically arranged to group them into new and consistent response options for both countries, in the same way as shown in Figs. 1 and 2.

**Loneliness** and **social isolation** as *outcome variables* were categorized, according to available measures in each country (see Figs. 1 and 2).

*Loneliness* indicator in Latvia was measured with a question “How often do you feel lonely” and “Would you say that you are lonely?” in Iceland.

*The Social isolation* variable for the Latvian population was constructed from 4 outcomes by social contact frequency with: “own children”, “own parents”, “other relatives”, “neighbours, friends or colleagues” (Fig. 2).

In the results chapter (see Figs. 7, 9 and 12) *Social isolation* have been renamed to integrate the Icelandic and Latvian datasets. The following changes to the Icelandic variables on social isolation were made by pulling from two composite variables: 1) Do you think you became socially isolated after the start of the COVID-19 epidemic? and 2) Do you still feel socially isolated? For the binary variable for social isolation, those who experienced rather little social isolation, or very little or no social isolation, belong in the category 1 *(not socially isolated)*. Those who experienced very much social isolation, rather much social isolation, or neither much nor little social isolation belong in the category 2 *(socially isolated)*. Further, the *social isolation* variable was constructed from two questions, one asking if you were isolated after COVID-19 began, and the other whether you still feel socially isolated.

### Categorisation of other variables

**Figure 3:**
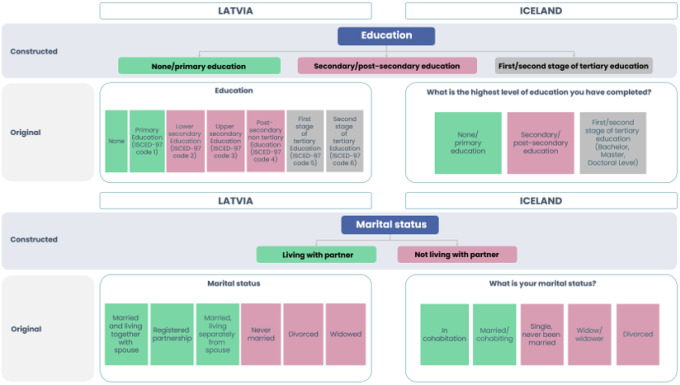
Socio-demographic factors

**Figure 4:**
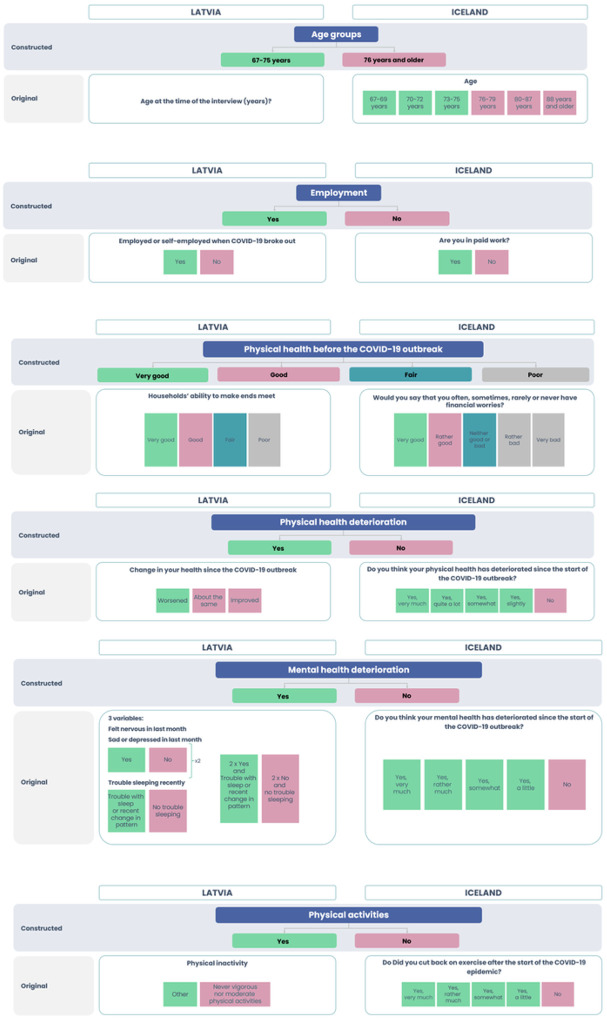
Physical and mental health, physical activities

Please refer to figure 3 and 4.

### Statistical analysis

The data was analysed in IBM SPSS 27 and Jamovi v. 2.3.18. Descriptive statistics as well as the Pearson Chi-square test were used to compare the differences between Latvia and Iceland and within each country.

## Results

**Figure 5:**
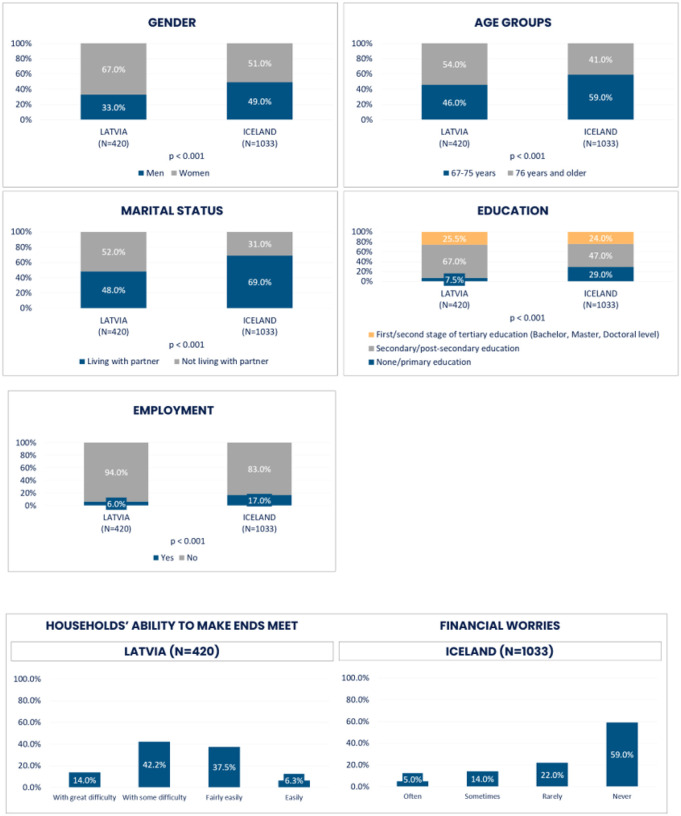
Comparison of demographic and socioeconomic variables between Latvia and Iceland

The data on the populations of age 67 + in Latvia and Iceland differed significantly across all studied demographic variables (Fig. 5). The proportion of older women was considerably higher in Latvia (67%) compared to a rather equal distribution of gender in Iceland. The proportion of Icelandic respondents in the younger group, 67–75 years, was significantly larger (59%) compared to Latvians (46%). Also, within the two countries, more than a half of Latvian respondents (54%) were in the age group of 76 years and older, while in Iceland, only 41%. It was more common to live with a partner in Iceland (69%) compared to Latvia (48%). Education level seemed to be somewhat higher among the respondents in Latvia, but there were more employed persons of age 67 + in Iceland (17%) compared to Latvia (6%). Generally, respondents in Iceland reported a considerably better financial situation compared to their Latvian counterparts. While only 6.3% of the respondents in Latvia answered that their household has the ability to make ends meet, 69% of the Icelanders did not experience financial worries.

### Comparison of loneliness and social isolation in Latvia and Iceland

**Figure 6:**
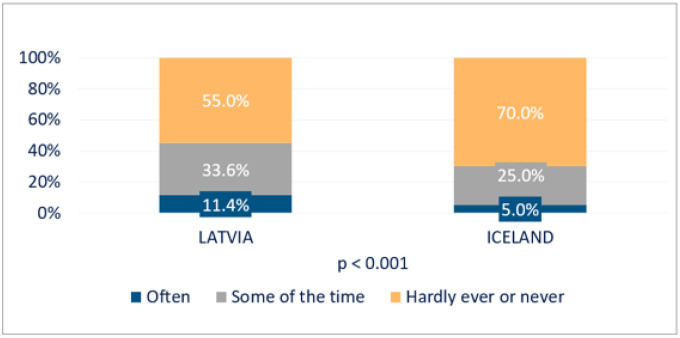
Loneliness among persons of age 67+ in Latvia and Iceland.

The Latvian older population reported considerably higher levels of loneliness compared to Icelanders (Fig. 6). Among Icelanders, 84% responded that they hardly ever or never experienced loneliness compared to 55% of Latvians. Latvian respondents were lonely more often (11.4%) than their Icelandic counterparts (5%).

**Figure 7:**
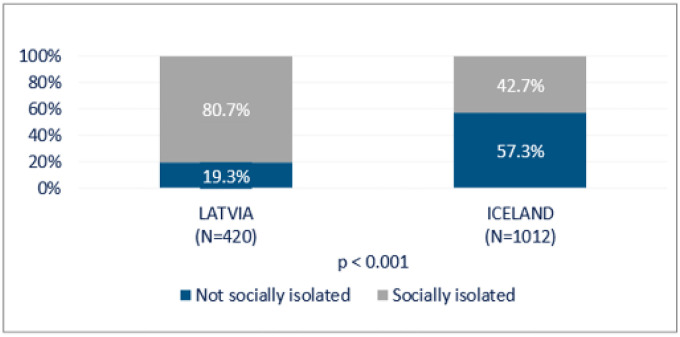
Social isolation among persons of age 67+ in Latvia and Iceland

Consequently, Latvian respondents felt more socially isolated (80.7%) compared to Icelanders (57.3%) (Fig. 7). Thus, only one in five Latvian and two in five Icelandic respondents did not experience social isolation.

**Figure 8:**
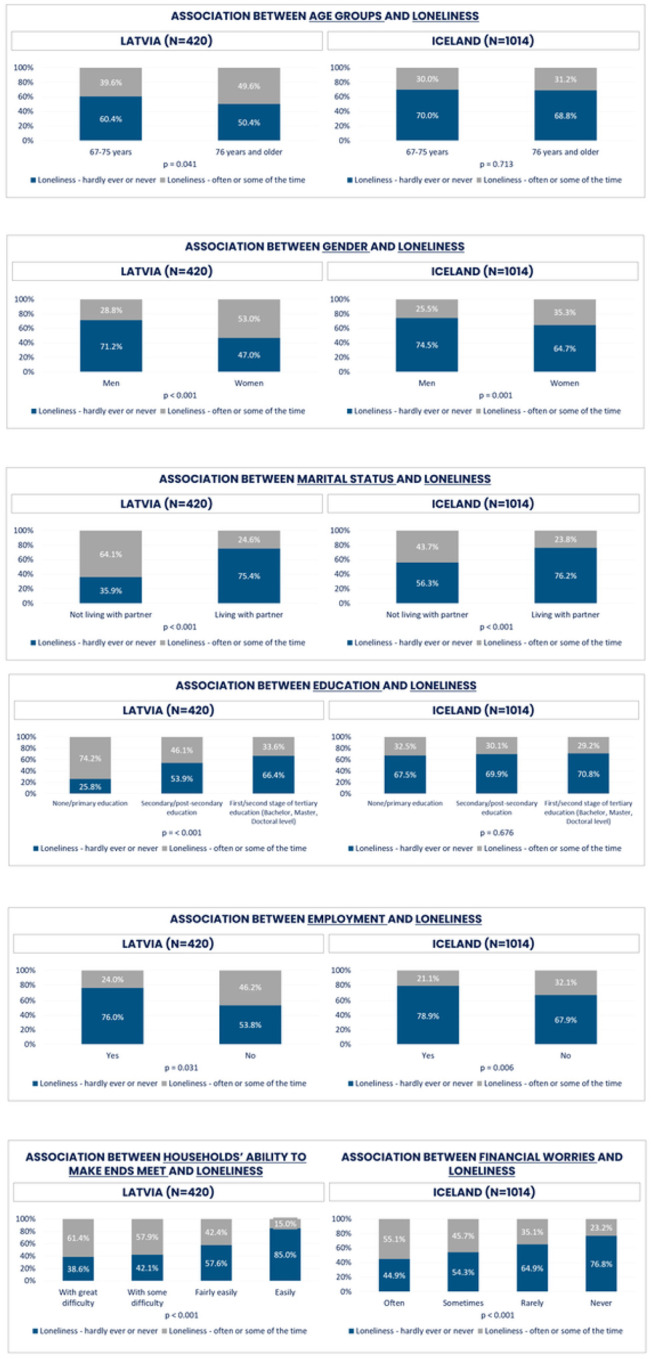
Loneliness and demographic and socio-economic factors for Latvia and Iceland (%)

**Figure 9:**
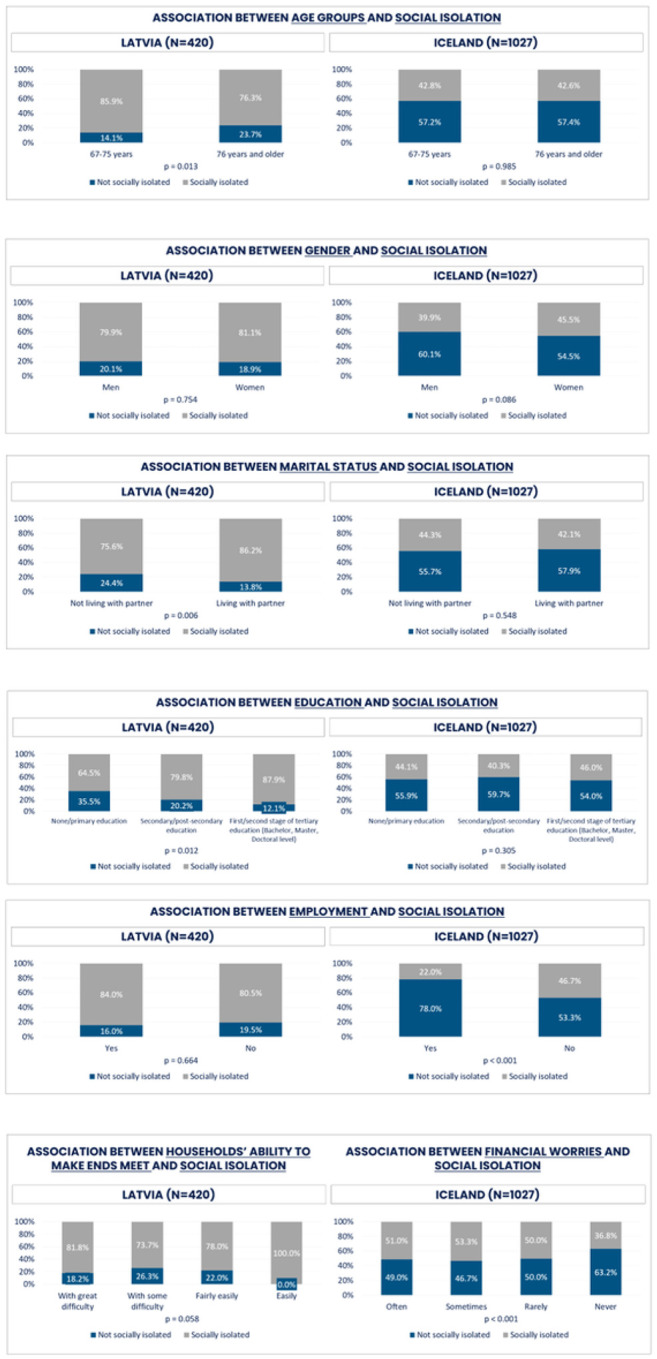
Social isolation and demographic factors for Latvia and Iceland (%)

Figure 8 shows the comparison of feeling lonely between Latvian and Icelandic population age 67 + in a number of demographic variables. In both Latvia and Iceland, women tended to experience loneliness more often compared to men. There were no significant differences between the age groups in Iceland and just a slight overrepresentation of lonely older Latvians. As expected, those living with a partner were less lonely, still approximately one in four felt lonely even when being in a partnership in both countries. Employment also seemed to be related to decreased loneliness. Those with higher education were significantly less lonely (46.1%), but educational attainment showed no differences in loneliness among Icelandic older adults.

Those with less financial worries were hardly or never lonely to a larger extent. Also, those who experienced more frequent number of visits by children, relatives and friends seemed to be less lonely. Phone contact, however, did not result in any significant differences in loneliness. The frequency of contacts on the internet and online communication platforms showed that several times a week was more common among those being less lonely.

Respondents between 67–75 years of age in Latvia seemed to experience more social isolation compared to Icelanders where no age difference was observed. It is worth noting that approximately 58% of Icelandic respondents were not socially isolated irrespectively age group in contrast to Latvians. Relatively similar experience of social isolation was between both genders in Latvia and Iceland.

**Figure 10:**
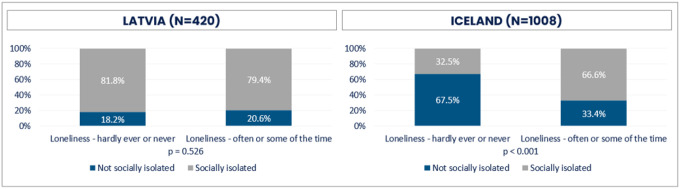
Loneliness and social isolation for Latvia and Iceland (%)

Four out of five Latvians responded that they were socially isolated (Fig. 10). There was no statistically significant difference in feeling socially isolated between those who either did or did not experience loneliness in Latvia. In Iceland, on the contrary, less lonely respondents did not experience social isolation to a considerably larger extent than those who felt lonely. Still, approximately one third of those Icelanders who were lonely, felt socially isolated (33.4%).

**Figure 11:**
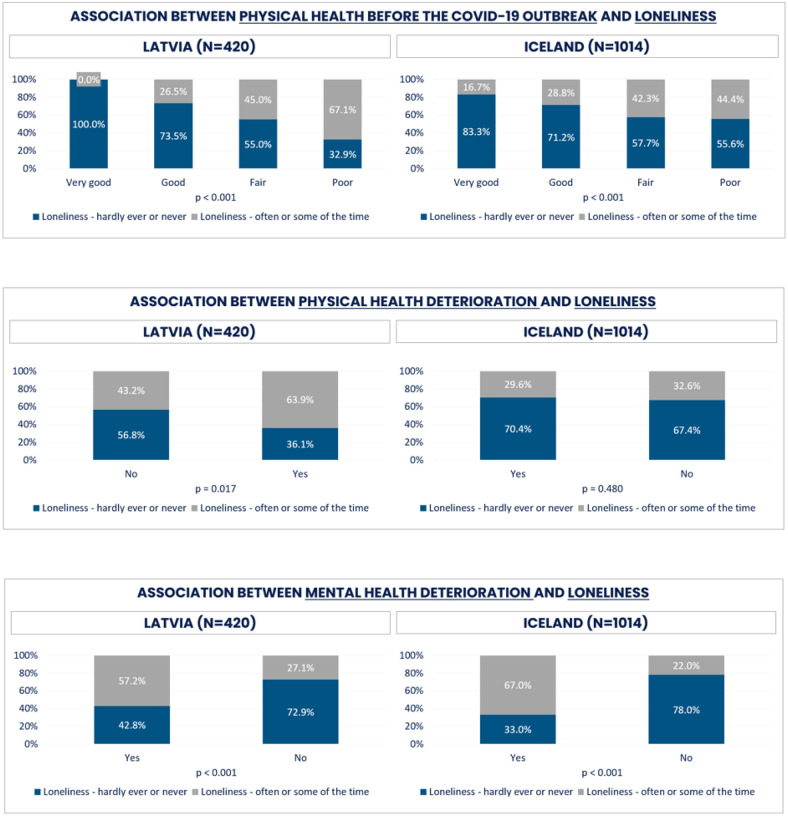
Loneliness and determinants for Latvia and Iceland

Significant associations were found between physical health before the COVID-19 outbreak and loneliness in both Latvia and Iceland (Fig. 11). None of the Latvian respondents who rated their physical health as very good before COVID-19 outbreak did report loneliness. In Iceland, however, 16.7% of respondents with very good health felt lonely. In both countries, those having poor, rather, or very poor physical health were lonelier, to a larger extent in Latvia (67.1%) compared to Iceland (44.4%). Differences in health status before the COVID-19 outbreak between lonely and not lonely groups were statistically significant for both Latvia and Iceland.

Those who were physically less active were also lonelier in Latvia (58.6%) compared to Icelanders, whose physical activities were not significantly different among the lonely persons.

Significant differences in mental health deterioration were found in both Latvia and Iceland. Among lonely respondents there was a considerably larger proportion of those reporting mental health deterioration (57.2% respective 67.0%).

No statistically significant differences were found in any of the health determinants for social isolation in Latvia between those reporting as being socially isolated or not. However, when comparing isolated groups with non-isolated, not socially isolated respondents were generally better off. In Iceland, however, all health factors were considerably different across response alternatives. Not socially isolated respondents tended to report better physical health before the COVID-19 outbreak, as well as less or no physical and mental health deterioration compared to socially isolated persons.

## Discussion

Latvian respondents aged 67 + reported to be lonelier compared to their Icelandic counterparts. While both Latvia and Iceland have been affected by social isolation due to the COVID-19 pandemic, different levels of social isolation were found, with Iceland having lower social isolation compared to Latvia. Four in five elderly Latvian respondents experienced higher social isolation (80.7%), compared to around half of the proportion who felt lonely often or some of time (45%). Among Icelanders, social isolation was much lower compared to Latvians (42.7%), and also a considerably lower proportion felt lonely (30%).

**Figure 12:**
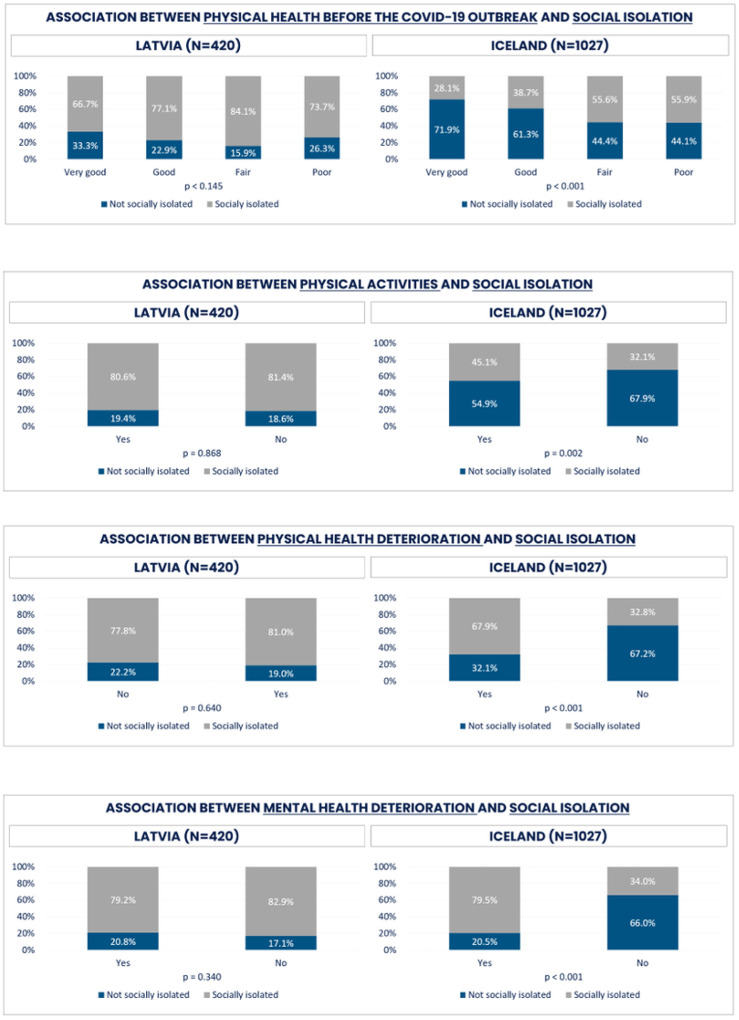
Social isolation and determinants for Latvia and Iceland

The results indicated physical and mental health deterioration during the COVID-19 pandemic. Strong deteriorating effects on mental and physical health was found among both lonely Latvian and Icelandic respondents (Fig. 11). However, deterioration in health was stronger for more socially isolated Icelanders compared to Latvians (Fig. 12). We identified several outstanding factors lying behind our findings in both countries, besides potential impact of cultural, geographic, and population characteristics.

Women were generally lonelier compared to men, which is in line with findings in other studies [12]. Loneliness also tended to be more frequent among the oldest group (REF). Social isolation exists among both men and women in the studied age groups in both countries, but was higher among those who lived with a partner. Earlier studies have pointed out that partnership does not guarantee social connectedness, and can depend both on the quality of social contacts as well as access to social and community resources [9, 19].

Also, those with a lower level of education in Latvia were lonelier, but this difference related to education did not appear in Iceland. In Iceland, there was a difference in the frequency of social isolation in relation to employment status, where a lower percentage of those who felt isolated were employed. These results require further examination, as different methods and resources are needed when dealing with social isolation or loneliness.

Differences in loneliness and the levels of social isolation might partly be explained by the demographic factors. First of all, the respondents in the Icelandic sample were younger than Latvian respondents, and, thus, to a larger extent in the labour market. Furthermore, the population of Latvia is overrepresented by elderly women, and they are more often living in single-person households, which puts them at a higher risk for both loneliness and social isolation [17]. Only about 48% live with a partner in Latvia compared to 69% in Iceland. This proportion is found to be particularly high for lone elderly women in Italy, France, Spain, Hungary and Latvia, while it is lowest in the Nordic countries [32]. Thus, the risk of isolation, or, in other words, the share of women who have felt lonely, is rather high (53%) among elderly lone women in Latvia; more than twice that of the elderly women living in couples (see Fig. 11). The equal distributions across both genders at age 67 + and a higher proportion of cohabiting persons in Iceland also might explain the lower levels of loneliness and social isolation. Therefore, it is likely that increased loneliness might be explained by emotional and existential factors, i.e. the loss or absence of important relationships and the experience of not belonging to a group or family, especially for elderly women. Long-term exposure to either protective or risk factors can become stronger as individuals age [9], and those elderly single women in Latvia might run a higher risk of loneliness. In the two studied populations, the effects of social disconnection (neglect, strain, isolation) or connection (supportive, stable family environment) cannot be directly measured, but may affect the feelings of loneliness and social isolation in later life, and might be related to the community ties, culture and traditions. Further, a number of other important life transitions, like retirement, children leaving home, age-related health problems, widowhood, especially among older women, may result in disruptions or decreases in social connection, but not measurable in this study. Thus, more gender analyses are needed to study the most exposed groups, e.g. older single women who constitute the majority of the oldest population in Latvia. There is a need to pay special attention and possibly urgent action to assess the situation of these women. It is also worth noting that, although it can be assumed that age groups decrease with increasing age, this is not the case in Latvia, explained by a large group of women who are over 85 [33].

The absence of financial worries seemed to be a protective factor against loneliness, which can be explained by easier access to social and cultural activities that require financial resources. A small proportion of Icelandic senior citizens had financial concerns compared to Latvians. Again, this may be explained by the composition of the group, i.e. overrepresented by elderly women, and also by gender pay gap and a non-favourable social security system for women in Latvia. On the other hand, higher education was more common among less lonely persons in Latvia, implicating that not only the professional networks and higher income, but also cognitive functioning are significant interacting factors [34]. Those with wider social networks were generally less lonely, and the utilisation of new online communication platforms seemed to be a protective factor, as opposed to the use of a phone to communicate with relatives and friends, that did not seem to be a protective factor at all.

This situation also calls for a closer look at what factors in each country influence or can explain the situation of those who are not socially isolated or lonely - that is, the influencing factors of healthy ageing.

The long-lasting effect of COVID-19 on social isolation among the elderly is likely to be significant mostly for the Icelandic population, while the loneliness feelings were common for both Latvian and Icelandic elderly populations (Figs. 11 and 12). The pandemic has disrupted normal social activities and resulted in increased isolation for many older people, particularly those who are at higher risk for severe illness and death from the virus. This can lead to feelings of loneliness and depression, which can have a negative impact on overall health and well-being [8]. It is important for communities to work together to find ways to support older adults and reduce their social isolation during and after the pandemic. This can include offering virtual social opportunities, providing transportation to essential appointments and errands, and promoting regular communication and connection with friends and family.

In addition, efforts should be made to address the underlying factors that contribute to social isolation, such as access to technology, financial stability, and physical mobility.

It is important for communities to support and promote social engagement and connection among older adults. This can include providing opportunities for social activities, promoting intergenerational relationships, and addressing barriers to social participation, such as lack of transportation or financial security. By promoting social connection and addressing social isolation, we can support healthy aging for all individuals.

The results suggest that social isolation is a major factor in the loneliness of older people in both countries and supports previous findings that social isolation is a contributing factor and increases the risk of loneliness. Also, these results give a fairly clear picture that the social isolation of older people, both due to the COVID-19 epidemic and in general, is a subject that needs to be taken seriously because the increased risk of loneliness is a threat to the health and well-being of older people.

### Limitations of the study

It can be complicated to compare manifestations of phenomena like loneliness and social isolation in different settings that represent both studied countries, and the diversity of phenomena definitions and measurements has posed a challenge. Comparing data from different databases in Latvia and Iceland which include similar questions but with different response alternatives can lead to methodological limitations. The measures used in the Latvian and Icelandic databases may not be equivalent or have the same level of validity and reliability. This can lead to differences in responses that are not true differences in attitudes or behaviours. For instance, a score of 5 on a scale used in Iceland might be equivalent to a score of 3 on a scale used in Latvia. However, both theoretical and statistical assumptions have been tested to make the scales comparable as much as possible.

The cultural, political, and economic context of Iceland and Latvia might also be different. Therefore, the way in which people answer the same question may differ based on context as well as the complex concepts of language. Although we selected respondents by age groups, the sampling methods in Latvia and Iceland might differ in terms of the size, representativeness, and methods used. This can lead to differences in the characteristics of the sample, and therefore differences in responses. Overall, comparing data from different databases in Latvia and Iceland should be done with caution, but much effort was made to ensure that the data is comparable and valid for a model fitting for both countries.

## Conclusions

The study confirmed that there are differences between the two countries regarding the frequency of loneliness and social isolation, with more elderly people in Latvia describing problems than their peers in Iceland. The study suggests that social isolation is a major factor in the loneliness of older people in both countries and confirms previous findings that social isolation is a contributing factor and increases the risk of loneliness, which might be enhanced by restrictions set during the COVID-19 pandemic. It is important for governments, communities, and individuals to recognize the impact of loneliness and social isolation on the well-being of the elderly and to take proactive steps to support this population. This can improve the quality of life for elderly individuals and help them maintain their independence and connection to their communities. Social factors are affecting ageing processes in Latvia; however, older age and the effects of the COVID-19 pandemic put Icelandic respondents in a vulnerable position. Thus, prioritization of some groups should be made and actions taken should be related to income, access to services, transportation, attitudes in the society etc.

## Availability of data and materials

This study used data from the Survey of Health, Ageing and Retirement in Europe, which is freely available to academic researchers. http://www.share-project.org accessed on November 30, 2022. The use of the Icelandic data from HL20 is based on the consent from all funding. The use of Icelandic HL20 data is based on the approval of public bodies and other parties that originally organized and paid for the costs of the research.
